# Transcriptomics of coping strategies in free-swimming *Lepeophtheirus salmonis* (Copepoda) larvae responding to abiotic stress

**DOI:** 10.1111/mec.12072

**Published:** 2012-10-25

**Authors:** Ben J G Sutherland, Stuart G Jantzen, Motoshige Yasuike, Dan S Sanderson, Ben F Koop, Simon R M Jones

**Affiliations:** * Centre for Biomedical Research, Department of Biology, University of VictoriaVictoria, BC, Canada V8W 3N5; † Aquatic Genomics Research Center, National Research Institute of Fisheries Science, Fisheries Research Agency2-12-4 Fukuura, Kanazawa, Yokohama, Kanagawa 236-8648 Japan; ‡ Pacific Biological Station3190 Hammond Bay Road, Nanaimo BC, Canada V9T 6N7

**Keywords:** abiotic stress, copepod, ecological genomics, salinity, sea lice, transcriptomics

## Abstract

The salmon louse *Lepeophtheirus salmonis* is a marine ectoparasite of wild and farmed salmon in the Northern Hemisphere. Infections of farmed salmon are of economic and ecological concern. Nauplius and copepodid salmon lice larvae are free-swimming and disperse in the water column until they encounter a host. In this study, we characterized the sublethal stress responses of *L. salmonis* copepodid larvae by applying a 38K oligonucleotide microarray to profile transcriptomes following 24 h exposures to suboptimal salinity (30–10 parts per thousand (‰)) or temperature (16–4 °C) environments. Hyposalinity exposure resulted in large-scale gene expression changes relative to those elicited by a thermal gradient. Subsequently, transcriptome responses to a more finely resolved salinity gradient between 30 ‰ and 25 ‰ were profiled. Minimal changes occurred at 29 ‰ or 28 ‰, a threshold of response was identified at 27 ‰, and the largest response was at 25 ‰. Differentially expressed genes were clustered by pattern of expression, and clusters were characterized by functional enrichment analysis. Results indicate larval copepods adopt two distinct coping strategies in response to short-term hyposaline stress: a primary response using molecular chaperones and catabolic processes at 27 ‰; and a secondary response up-regulating ion pumps, transporters, a different suite of chaperones and apoptosis-related transcripts at 26 ‰ and 25 ‰. The results further our understanding of the tolerances of *L. salmonis* copepodids to salinity and temperature gradients and may assist in the development of salmon louse management strategies.

## Introduction

The salmon louse *Lepeophtheirus salmonis* (Copepoda: Caligidae) is an ectoparasite of wild and farmed salmonids (*Salmo* and *Oncorhynchus* spp.) in the Northern Hemisphere (Nagasawa *et al*. 1993; Johnson *et al*. 2004; Beamish *et al*. 2009), although genetically distinct varieties of *L. salmonis* occur in the Atlantic and Pacific Oceans (Yazawa *et al*. 2008). The louse develops through three free-living and nonfeeding stages (nauplii I and II and the infective copepodid) and seven parasitic stages (four nonmotile chalimus, two motile pre-adult stages and one motile adult) (Johnson & Albright 1991). In British Columbia, Canada, adult Pacific salmon carry gravid *L. salmonis* when they return from the ocean to spawn (Beamish *et al*. 2005). In addition, farmed salmon in open-net pens and other resident hosts in the area support infections with the parasite (Johnson *et al*. 2004; Morton *et al*. 2004; Beamish *et al*. 2005; Jones 2009). If not properly managed, infections transmitted from farmed salmon can cause epizootics on juvenile wild salmon leading to population-level effects (Krkošek *et al*. 2007). The costs of treatment and management of *L. salmonis* on farmed salmon globally are approximately $400M CAD per annum; infections remain a major obstacle to sustainable industry development (Costello 2009). There are a limited number of chemical treatment options (Johnson *et al*. 2004), raising concerns for resistance development to commonly used treatments in Scotland, Norway and Atlantic Canada (Jones *et al*. 1992; Denholm *et al*. 2002; Boxaspen 2006; SEARCH 2006; Brooks 2009; Burridge *et al*. 2010; Chang *et al*. 2011). Integrated pest management principles advocate reduction of pesticide reliance to avoid resistance development and minimize environmental residues (Brooks 2009; Burridge *et al*. 2010). Other potential methods of control may include the use of cleaner wrasse, leaving farms to fallow, reducing synthetic light, and ensuring high water velocity at sites (SEARCH 2006).

The biology of the salmon louse is strongly influenced by environmental conditions, and there is an interest in understanding how changes in these conditions affect the propagation dynamics of louse populations (Brooks 2005, 2009; Price *et al*. 2010). For example, temperature influences fecundity and time to hatching (Boxaspen & Næss 2000; Johnson *et al*. 2004; Boxaspen 2006; Costello 2006), and increased temperature during exposure results in increased louse settlement success, development and prevalence over a 10-day experimental infection (Tucker *et al*. 2000). Development and survival of *L. salmonis* are optimal at salinities greater than 26 parts per thousand (‰) (Bricknell *et al*. 2006). Without a host, adult female *L. salmonis* can osmoregulate down to 12.5 parts per thousand (‰) salinity (<8 h to death in freshwater), while adult lice attached to the host survive in freshwater from 3 to 7 days, possibly through diet-obtained ions (Hahnenkamp & Fyhn 1985; Connors *et al*. 2008). Experimental infections of Atlantic salmon with copepodids at 34‰ or 24 ‰ consistently resulted in reduced settlement success and slower louse development at 24 ‰ (Tucker *et al*. 2000). In contrast to attached stages, larval lice are more sensitive to low salinity, potentially due to the absence of dietary ions and the increased energetic demands of the hyposaline stress (Bron *et al*. 1993; Bricknell *et al*. 2006). Copepodid development is inhibited at salinities <30 ‰ (Johnson & Albright 1991), although detrimental effects may be transient if exposure is short term (Bricknell *et al*. 2006). Experimental incubations suggest negative effects on copepodids are manifested at salinities <27 ‰: several hours at ~26 ‰ severely compromised survival and infectivity potential; 1 h at 16 ‰ resulted in mortality of approximately 50% of copepodids; and below 12 ‰, death was rapid (Bricknell *et al*. 2006). An improved understanding of the larval *L. salmonis* response to hypo-osmotic environments may allow the incorporation of salinity levels into parasite management strategies (Brooks 2009).

The application of genomics to copepod biology provides ecological, evolutionary and economic insights (Bron *et al*. 2011) and adds to the knowledge base from ecotoxicology studies (Raisuddin *et al*. 2007). Recently, a transcriptomic analysis of hyposaline responses in the euryhaline green crab *Carcinus maenas* has provided new information on the responses of crustaceans to environmental salinity changes (Towle *et al*. 2011). Many gene expression studies of environmental abiotic stressors in marine copepods (temperature, salinity, environmental contaminants) utilize specific gene markers and enzyme isoforms (Lauritano *et al*. 2011), although transcriptomic studies exist (*e.g. Tigriopus japonicus* responses to copper; Ki *et al*. 2009). Collectively, these studies indicate large variations in responses, but identifying stress-specific markers remains a goal (Lauritano *et al*. 2011). Transcriptomics has also been applied to identifying genes involved in *L. salmonis* postmoulting maturation and egg production (Eichner *et al*. 2008). The earlier observations support a hypothesis that *L. salmonis* experiences physiological stress in association with reduced salinity and that this depends on salinity level, development stage and host association. The development of a 38K *L. salmonis* oligonucleotide microarray described herein has provided a platform to test this hypothesis and to characterize the transcriptomic basis of the stress response of free-swimming *L. salmonis* responding to changes in environmental salinity or temperature.

## Methods

### Animal preparation, exposures and RNA extraction

*Lepeophtheirus salmonis* obtained from seawater netpen-reared Atlantic salmon *Salmo salar* in western British Columbia were maintained in cold aerated seawater during transport to the Pacific Biological Station, Nanaimo, BC. Intact and pigmented egg strings were removed and incubated in flasks containing 400 mL of filtered and aerated seawater. The resulting nauplii were maintained at 30 ‰ salinity until a majority moulted to copepodids (Johnson & Albright 1991), at which time they were pooled and then aliquoted into groups of ~500 lice per beaker. Triplicate flasks were incubated for 24 h at 4, 10 or 16 °C with salinity held constant at 30 ‰. In another experiment, triplicate flasks containing seawater diluted to 30 ‰, 25 ‰, 20 ‰ or 10 ‰ were incubated at 10 °C. These wide-range experiments were repeated once. A single high-resolution salinity experiment was conducted as above, but with six beakers per condition and at salinities of 30 ‰, 29 ‰, 28 ‰, 27 ‰, 26 ‰ and 25 ‰ and a constant temperature of 10 °C.

The lice were recovered onto 47-mm cellulose acetate/cellulose nitrate filter membranes with a pore size of 8.0 μm (EMD Millipore). The membranes were flash-frozen in liquid nitrogen and stored at −80 °C. Frozen filters containing lice were homogenized with a mixer mill (Retsch® MM 301), and RNA was extracted using TRIzol® (Invitrogen), as per manufacturers' instructions, and purified through RNeasy spin columns with an on-column DNase I treatment (QIAGEN) to degrade genomic DNA. Total RNA was then quantified by spectrophotometry (NanoDrop-1000) and quality-checked by electrophoresis on a 1% agarose gel. Samples were then randomized for all downstream nucleic acid manipulations.

### cRNA synthesis and reference pool generation

Purified total RNA (200 ng) was reverse-transcribed to cDNA and then transcribed to labelled cRNA using Low Input Quick Amp Labeling kits (Agilent), as per manufacturer's instructions for hybridization to a 4-pack oligo gene expression microarray. Labelled cRNA was purified through RNeasy columns as per manufacturer's instructions (QIAGEN) and quantified using spectrophotometry (NanoDrop-1000), ensuring specific activity of all samples >6 pmol dye per microgram cRNA (Agilent). Samples were kept at −80 °C until hybridization. A reference pool of Cy3-cRNA was synthesized by amplifying experimental samples as described previously, but with Cy3-CTP-labelled nucleotide (Perkin Elmer). For each experiment, a reference pool was generated using equimolar cRNA from each experimental condition. In the wide-range salinity experiment, the 25 ‰ condition was added at a later date, and therefore, this condition was not included in the reference.

### Microarray hybridization, quantification, normalization and filtering

A 38K oligo microarray was designed using previously annotated ESTs from both Pacific and Atlantic *L. salmonis* (Yasuike *et al*. 2012) using eArray (Agilent) with selection of probes preferentially at 3′ untranslated regions. Sample and reference combinations (825 ng cRNA each) were fragmented then hybridized at 65 °C for 17 h at 10 rpm as per manufactures' instructions (Agilent) using SureHyb chambers (Agilent). Washing was performed as per manufacturers' instructions, using the optional protocol to prevent ozone degradation. All slides were transferred to a dark box and kept at low ozone until scanned on a Perkin Elmer ScanArray® Express at 5 μm resolution using PMT settings optimized to have the median signal of ~1–2% of array spots saturated (Cy5: 70; Cy3: 70).

Images were quantified in Imagene 8.1 (Biodiscovery) using an eArray GAL file (Design ID: 024389; Agilent). Poor spots and control spots were flagged by the software for downstream filtering. A block-specific background correction was performed by subtracting the average median signal for negative control spots from each signal median. Sample files were loaded into GeneSpring 11.5.1 (Build 138755; Agilent) and have been uploaded to GEO (GSE37976). Each experiment was normalized and filtered separately as follows: raw value threshold of 1.0; intensity-dependent *Lowess* normalization; and baseline transformation to the median of all samples. Control spots and any probes not passing the following filter were removed from the analysis: raw values ≥500 in at least 65% of samples in any one condition and no flags in at least 65% of samples in any one condition.

### Differential expression and functional analysis

Array probes were tested for significance in each experiment using a one-way anova without equal variance assumption, with a *post hoc* Tukey's HSD (*P* ≤ 0.01). Probes were filtered for fold change difference ≥1.5 from control (10 °C and 30 ‰ in temperature and salinity experiments, respectively). All probes passing significance and fold change filtering in the salinity experiment (high resolution) were used as an input for K-means clustering (Euclidean distance metric; 5 clusters; 50 iterations; GeneSpring 11.5.1 Agilent). Gene ontology (GO) and pathway enrichment were performed in DAVID bioinformatics tool (modified Fisher's exact test; Huang *et al*. 2009), using Uniprot accession numbers of clustered probes compared with a background list as all probes passing quality control filters for each experiment.

### Reverse transcriptase–quantitative polymerase chain reaction (RT–qPCR)

The same RNA samples used for microarrays in the high-resolution salinity experiment were used for RT–qPCR. Synthesis of cDNA was performed with 2 μg total RNA in 20-μL reactions using oligo(dT) primers and SuperScript III First-Strand Synthesis System for RT–PCR (Invitrogen), as per manufacturer's instructions. Each cDNA sample was diluted 20-fold. To generate a standard curve, one sample from each of the six conditions was randomly selected and synthesized as described previously. These samples were then pooled and diluted 7-fold. This pool was then used for a serial dilution (5-point, 5-fold each point) for efficiency tests. qPCR amplification was performed using SsoFast™ EvaGreen® (Bio-Rad) in 20-μL reactions with 0.3 μm of each primer using the following thermal regime: segment 1, 95 °C for 30 s, 1 cycle; segment 2, 95 °C for 5 s, 55 °C for 20 s, 40 cycles; segment 3, 95 °C for 10 s; melt curve, ramp from 55 to 95 °C (fluorescence read each 0.5 °C increment). Genes of interest were selected from the microarray results due to biological relevance, high significance level or presence in significantly enriched GO categories. Reference gene candidates were selected from microarray results indicating stable expression across conditions, consistency across replicate spots and moderate levels of expression as well as from previous literature (Frost & Nilsen 2003). Primers were designed in Primer3 (Rozen & Skaletsky 2000) selecting amplicon sizes of 80-150 base pairs (Table 1; all *R*^2^ were ≥0.99)). Amplicons were checked for single products by melt curve analysis and were sequenced to confirm identity as previously described (Sutherland *et al*. 2011).

**Table 1:** Primers used for RT–qPCR, product sizes and efficiency values

Gene	Sense primer	Antisense primer	Size	Eff.%
Chromobox protein homolog 1 (*cbx1*)	TCATTGGAGCCACAGATTCC	TCACTGTTTGAGGACATCGC	117	99
Chromobox protein homolog 2 *(cbx2)*	CAAATGCCACCAATCTCTCC	CATCGTGATCAAATTCACCG	118	111
Histone-binding protein RBBP4 *(rbbp4)*	GAGAAGTGAATCGTGCTCGG	CACGAGAACATCAGAGCTGG	80	97
Heat shock protein HSP 90-alpha *(hsp90aa1)*	CGGGATAACTCAACTGTCGG	CATTCTTGTCAGCATTTGCC	109	93
T-complex protein 1 subunit zeta *(cct6)*	CATGAAGGCTGCCAATAAGC	ACTTCAAAAGCTCCAGCACC	123	97
Protein disulphide isomerase A3 *(pdia3)*	CCCATCTACGAGGAACTTGG	GGAACATCATTTGCCGTAGC	83	101
Calreticulin *(crt)*	CGACCCTGAAGCATCTAAGC	CATTTACCCTTGTATGCGGG	138	103
Apoptosis-stimulating of p53 protein 2 *(tp53bp2)*	GGACTCCTCTTCATTGTGCC	AACCATGAAAGCCTTCCTCC	150	116
Programmed cell death protein 4 *(pdcd4)*	TCAATCGTAAGATGCCGTCC	CCAGTATTCCTTGAATCGGC	77	105
Growth arrest-specific protein 1 *(gas1)*	GTGAGGAACAGGAAACAAATCC	ACAACATCCGTTTCACCTCC	106	105
Adenine phosphoribosyltransferase *(aprt)*	GTTGAGGAAAAAGCATTGCC	TTGGAACAAAAGGAACTCCG	118	111
GTP-binding protein SAR1b *(sar1b)*	GTCCAGTTCTCATTTTGGGC	CCTTTCCCGGTAGTTTGACC	103	102
FK506-binding protein 4 *(fkbp4)*	ATGGTTCCCAAAGAAGAGGC	ATCGCTCTTTGGAGTGTTCC	145	95
Myosin heavy chain, muscle *(mhc)*	GGAACTCACTTATGCCACGG	TTTGCTTCTTGTAGGAGCGG	101	90
High-affinity copper uptake protein 1 *(slc31a1)*	CTACAAATCCCACTGAATGCC	AATTGAAGGACGTGCAGAGC	106	102
Structural ribosomal protein S20 *(rps20)*	GTCACCTCAACCTCCACTCC	TGACTTGCCTCAAAGTGAGC	274	94
Glutathione S-transferase 1, isoform D *(gstd1)*	GGAGCTCCAACAACTTCAGC	AAGGAAGCTCTCTCGCACC	115	101
Tubulin beta chain *(tubb)*	TGCGGCTATATTTAGAGGGC	AGGTGGAATGTCACAAACGG	136	110
Vinculin *(vcl)*	AGATTCCAACACTGGGAACG	CAGAGTCCATTTTTGCTCCC	78	105

RT–qPCR data analysis was performed using qbasePLUS (Biogazelle). Stability of reference genes was tested using geNorm (Vandesompele *et al*. 2002). Selected reference genes included the previously identified gene *structural ribosomal protein S20* (Frost & Nilsen 2003) and *filamin-A*, with a collective M value of 0.581 and CV of 0.203, a value within the range typically observed for stably expressed reference genes in heterogeneous samples (Hellemans *et al*. 2007). Other tested reference genes that were not used to normalize due to higher variability included the following: *vinculin* and *tubulin beta chain* (*data not shown*). Technical replicates were within 0.5 Ct for 934/936 sample–target combinations. NTC and RT controls showed no amplification. Statistical significance was identified by one-way anova (*P* ≤ 0.05) with pairwise significance determined by means of confidence intervals (Biogazelle). Correlation between methods (RT–qPCR and array) were checked using a linear best fit lines of log_2_ expression values for RT–qPCR samples vs. microarray log_2_ expression ratios (Cy5/Cy3) for the probe corresponding to the contig used for primer design.

## Results

### Broad survey – responses to thermal and hyposalinity exposures

**Fig 1:**
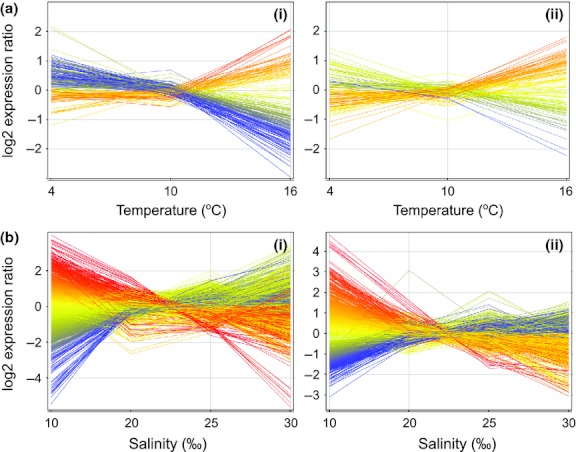
A 24 h exposure to changes in temperature (a) affected the expression of fewer genes compared with a similar exposure to hyposalinity (b). Each coloured line displays the average log_2_ expression ratio (Cy5-sample/Cy3-reference) of a transcript across all conditions. Each transcript is normalized to the median expression level of that transcript across all conditions. Each unit of vertical deflection of the expression ratio corresponds to a 2-fold change in regulation. Lines are coloured according to the magnitude and direction of expression at 10 ‰ or 16 °C. Each plot represents an independent experiment. To be present on a plot, a transcript must be differentially expressed in at least one condition compared with the control (one-way anova and Tukey's HSD *P* ≤ 0.01; FC ≥ 1.5).

Exposures to a wide range of salinity (10–30 ‰) and temperature (4–16 °C) were used to survey for transcriptome perturbances in *L. salmonis* copepodids. Temperature incubations for 24 h resulted in few genes differentially expressed from the 10 °C control (Fig. 1, Table 2). Total gene numbers differentially expressed (Table 2) and the fold change differences from the control (Fig. 1) suggested 16 °C had a greater influence on gene expression than did 4 °C. In general, few differentially expressed genes and low consistency between temperature exposure trials indicate that the temperature range selected does not have a strong effect on copepodid gene expression over a 24 h exposure.

**Table 2:** Overview of genes differentially expressed in response to temperature and salinity changes. 24 h exposure to hyposalinity resulted in a large number of differentially expressed genes, whereas temperature had less of an effect. Trials 1 and 2 represent independent experiments. Genes were tested for differential expression from control by one-way anova and Tukey's HSD (*P* ≤ 0.01; FC ≥ 1.5). Numbers of genes differentially expressed represent genes with unique annotations

Experiments	Comparison (°C or ‰)	Trial	Direction	Differentially expressed genes	Common bw. trials
Temp (WR)	4 vs. 10 °C	1	Up	13	1
		2	17
		1	Down	4	0
		2	12
	16 vs. 10 °C	1	Up	29	1
		2	19
		1	Down	38	5
		2	25
Salinity (WR)	25 vs. 30 ‰	1	Up	295	31
		2	59
		1	Down	119	6
		2	24
	20 vs. 30 ‰	1	Up	281	35
		2	97
		1	Down	139	9
		2	32
	10 vs. 30 ‰	1	Up	441	91
		2	209
		1	Down	340	72
		2	183
Salinity (HR)	29 vs. 30 ‰	n/a	Up	4	n/a
		Down	8
	28 vs. 30 ‰	n/a	Up	16	n/a
		Down	31
	27 vs. 30 ‰	n/a	Up	193	n/a
		Down	282
	26 vs. 30 ‰	n/a	Up	221	n/a
		Down	138
	25 vs. 30 ‰	n/a	Up	464	n/a
		Down	408

WR, wide-range experiment; HR, high-resolution experiment.

Hyposalinity exposures resulted in many genes changing in expression from the 30 ‰ control (Fig. 1). At 10 ‰, transcriptome perturbance was largest (Table 2), although many genes had already changed between 30 ‰ and 25 ‰. A larger number of differentially regulated genes were observed in salinity trial 1 relative to trial 2, and this difference may be from different copepod broods being used in each trial (Table 2). However, responding genes common to both trials were identified (see ‘*Common bw. trials*’ in Table 2), and these genes are presented in Table S1 (Supporting information). On average, the proportion of up-regulated genes shared between trials in each trial was approximately 15 % and 44 % for trial 1 and 2, respectively. The magnitude of fold changes for each differentially expressed gene, and the larger number of differentially expressed genes in the short-term hyposalinity exposure contrasts with the results of the short-term temperature exposure and indicates the importance of salinity for free-swimming *L. salmonis*.

Increased transcription of chaperones is often viewed as an indicator of cellular stress (Lauritano *et al*. 2011). Several chaperone or proteasome genes were up-regulated in both salinity trials (Table S1, Supporting information) including *26S proteasome non-ATPase regulatory subunit 6* (25 ‰), *26S proteasome non-ATPase regulatory subunit 4* (20 ‰, 10 ‰)*, proteasome subunit beta type-3* (10 ‰), *60-kDa heat shock protein, mitochondrial* (20 ‰, 10 ‰) and *heat shock 70 kDa protein cognate 4* (20 ‰). *Trypsin-1* was down-regulated in both trials at 25 ‰. *Programmed cell death protein 4* was up-regulated in both trials at 20 ‰ and 10 ‰. Interestingly, several cuticle proteins were up-regulated in both trials at 10 ‰ (*cuticle protein 6; cuticle protein CP14.6; chitin bind 4*). *Calreticulin* was identified as up-regulated in both trials at 10 ‰. The consistent presence of these chaperone- and apoptosis-related transcripts probably indicates hyposalinity stress in the copepodids.

### High-resolution profiling of hyposaline transcriptome responses

**Fig 2:**
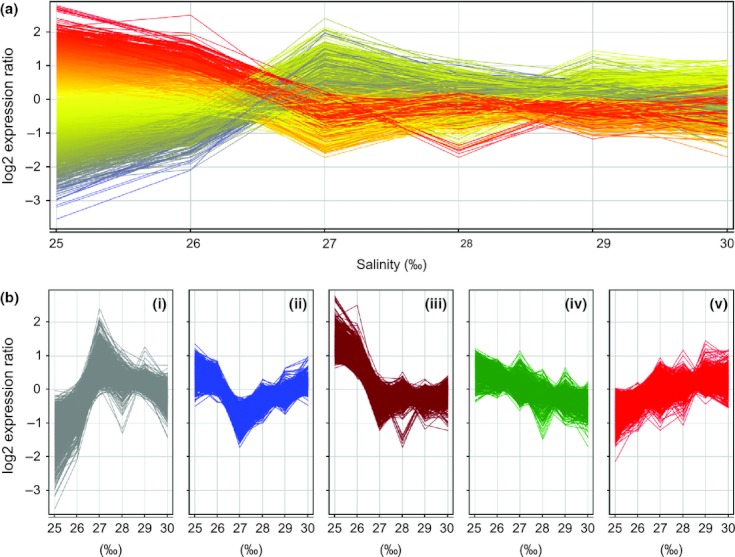
Gene expression affected by single unit changes in salinity between 25 ‰ and 30 ‰. (a) Overview of log_2_ expression ratios (Cy5-sample/Cy3-reference) of all transcripts differentially expressed from the control (in at least one condition) indicates few changes at 29 ‰ or 28 ‰, an initial response at 27 ‰, and a large secondary response at 26 ‰ and 25 ‰. Each transcript is normalized to the median expression level of that transcript across all conditions. Each unit of vertical deflection of the expression ratio corresponds to a 2-fold change in regulation. (b) Five patterns of expression were identified by cluster analysis, indicating different responses typical of different salinity levels. Differential expression was detected by one-way anova and Tukey's HSD (*P* ≤ 0.01; FC ≥ 1.5).

To identify a threshold of response, and to capture primary responses to hyposaline stress, a higher-resolution range was used (30–25 ‰, single increment decreases). Relative to the control, the number of transcripts differentially expressed increased rapidly at 27 ‰ compared with changes at 28 ‰ or 29 ‰ (1179 probes differentially expressed by 27 ‰; uniquely annotated genes: 193 up- and 282 down-regulated; Table 2; Fig. 2a). This increase in differentially expressed genes may indicate a threshold of response above which reduced salinity does not have a measurable effect on the transcriptome. At 26 ‰, differentially expressed transcripts belonged to a different suite of genes; many genes that responded in the 27 ‰ condition were not up-regulated in the 26 ‰ or 25 ‰ conditions (Fig. 2a). The greatest number of differentially expressed transcripts in the high-resolution study occurred at 25 ‰ (2451 probes differentially expressed; uniquely annotated genes: 464 up and 408 down). The differentially expressed transcripts were clustered by similar patterns of expression to resolve several salinity response types (Fig. 2b) described as primary, differentially regulated at 27 ‰ (cluster *i* and *ii*); secondary, differentially regulated at lower salinity (26 ‰ and 25 ‰; cluster *iii*); or continual, gradually increasing or decreasing across the exposure conditions (cluster *iv* and *v*). Primary response genes are either at baseline in lower salinity conditions or drop below the baseline. These different clusters are largely composed of different genes and response functions at different salinity levels (probes present in each cluster are presented in Table S2).

Certain chaperone types were typical of specific salinity responses. For example, *hsp90 alpha*,*hsp70 protein 14*,*protein disulphide isomerase 2*, and several *chaperonin-containing t-complex protein (cct) subunits* were all up-regulated at the primary peak (Table 3; Fig. 2i). However, with the exception of *cct subunit epsilon*, none of these genes were differentially expressed in the 26 ‰ or 25 ‰ response conditions. *Heat shock protein beta-1*, several *hsp70* isoforms and some *DnaJ homologs* increased at the lower salinities (Table 3). CCT substrate was originally thought to be restricted to tubulin and actin and linked to cell cycle progression; however, it is now known to have broader specificity (Brackley & Grantham 2009). CCT subunits may be up-regulated during proliferation; however, CCT's role in abiotic stress handling was recently identified in cold hardiness of insects during diapause (Rinehart *et al*. 2007).While the precise role of CCT in the present study is not clear, the concerted regulation with the other chaperones suggests a role in the maintenance of cellular function (Table 4). Proteasome activity is also identified within the primary peak (proteasome complex; *P* = 0.001; Table 4).

**Table 3:** Genes involved in protein folding and degradation were affected by hyposalinity exposure relative to 30 ‰ control. Genes were tested for differential expression from the control by one-way anova and Tukey's HSD (*P* ≤ 0.01; FC ≥ 1.5). Fold change ratios are log_2_(experimental) – log_2_(control) with standard error. Value of + 1 = 2-fold up-regulation. Absent values indicate no significant difference from control

Gene	ProbeID	Salinity (‰)						
25	26	27	28	29
Protein Folding – production and maintenance of proper protein conformation						
Heat shock 70 kDa protein 14	C250R106	*–*	*–*	1.04 ± 0.19	*–*	*–*
Heat shock protein HSP 90 alpha	C252R026	*–*	*–*	1.49 ± 0.31	*–*	*–*
T-complex protein 1 subunit alpha	C213R139	*–*	*–*	1.22 ± 0.29	*–*	*–*
T-complex protein 1 subunit beta	C010R138	*–*	*–*	2.21 ± 0.54	*–*	*–*
T-complex protein 1 subunit delta	C198R114	*–*	*–*	1.48 ± 0.29	*–*	*–*
T-complex protein 1 subunit epsilon	C170R116	1.20 ± 0.24	*–*	1.41 ± 0.42	*–*	*–*
T-complex protein 1 subunit zeta	C191R120	*–*	*–*	1.81 ± 0.41	*–*	*–*
T-complex protein 1 subunit eta	C213R160	*–*	*–*	2.29 ± 0.51	*–*	*–*
Protein disulphide isomerase 2	C242R105	*–*	*–*	0.95 ± 0.22	*–*	*–*
Heat shock 70 kDa protein	C150R102	3.02 ± 0.37	2.78 ± 0.42	*–*	*–*	*–*
Heat shock 70 kDa protein 4L	C192R161	1.28 ± 0.31	1.25 ± 0.29	*–*	*–*	*–*
Heat shock 70 kDa protein cognate 4	C219R057	*–*	1.27 ± 0.23	*–*	*–*	*–*
Heat shock protein beta-1	C172R035	1.25 ± 0.27	1.27 ± 0.41	*–*	*–*	*–*
Heat shock protein homolog	C130R040	−0.96 ± 0.15	*–*	*–*	*–*	*–*
Protein disulphide isomerase A4	C124R001	*–*	−1.38 ± 0.45	*–*	*–*	*–*
Protein disulphide isomerase A6	C088R134	−1.58 ± 0.43	*–*	*–*	*–*	*–*
DnaJ homolog subfamily B member 4	C006R133	1.75 ± 0.29	1.45 ± 0.47	*–*	*–*	*–*
DnaJ homolog subfamily B member 6-A	C251R008	1.00 ± 0.23	0.93 ± 0.26	*–*	*–*	*–*
DnaJ homolog subfamily C member 1	C107R150	1.22 ± 0.21	*–*	*–*	*–*	*–*
DnaJ homolog subfamily C member 27	C123R120	−1.57 ± 0.23	*–*	*–*	*–*	*–*
Proteasome – degradation of unneeded or damaged proteins						
26S proteasome non-ATPase regulatory subunit 2	C229R164	*–*	*–*	1.77 ± 0.35	*–*	*–*
26S proteasome non-ATPase regulatory subunit 4	C262R145	*–*	*–*	1.61 ± 0.31	1.19 ± 0.33	*–*
26S proteasome non-ATPase regulatory subunit 7	C091R061	−1.23 ± 0.44	*–*	*–*	*–*	*–*
26S proteasome non-ATPase regulatory subunit 8	C060R115	−0.76 ± 0.14	*–*	*–*	*–*	*–*
26S proteasome non-ATPase regulatory subunit 10	C091R010	1.11 ± 0.20	*–*	*–*	*–*	*–*
Proteasome activator complex subunit 4	C161R067	0.92 ± 0.21	0.89 ± 0.26	*–*	*–*	*–*
Proteasome subunit alpha type-6	C048R139	−1.11 ± 0.17	*–*	*–*	*–*	*–*
Proteasome subunit beta type-1	C134R118	−0.65 ± 0.13	*–*	*–*	*–*	*–*
Proteasome subunit beta type-2	C133R004	−0.75 ± 0.17	*–*	*–*	*–*	*–*
Proteasome subunit beta type-3	C055R153	−1.08 ± 0.14	−0.79 ± 0.22	*–*	*–*	*–*
Proteasome subunit beta type-4	C155R060	−0.96 ± 0.18	*–*	*–*	*–*	*–*

**Table 4:** Selected enriched functional categories in the five hyposalinity response patterns (clusters i-v in Fig. 2b). The primary peak (i) and secondary response (iii) represent different mechanisms responding to different levels of hyposalinity. Significance of enrichment was tested by a modified Fisher's exact test

Cluster	Type	Gene Ontology term	Genes in cluster	*P*-value
(i) Primary peak	BP	Cell redox homoeostasis	9	0.0052
		Carbohydrate catabolic process	10	0.0022
		Metamorphosis	8	0.0236
	CC	Proteasome complex	10	0.0010
	MF	Chromatin binding	6	0.0351
(ii) Primary valley	BP	Retrograde vesicle-mediated transport, Golgi to ER	4	0.0108
		Electron transport chain	9	0.0128
	CC	Mitochondrion	57	1.29E-07
	MF	Structural constituent of ribosome	13	7.31E-04
	N-acetyltransferase activity	5	0.0450
(iii) Secondary response	BP	Transport	45	0.0086
		Response to stress	18	0.0378
		Small GTPase-mediated signal transduction	14	2.51E-06
		Wing disc development	6	0.0189
		Gamete generation	12	0.0184
MF	Small conjugating protein ligase activity	8	0.0283
(iv) Gradual up	BP	Macromolecule catabolic process	8	0.0141
		Proteolysis	9	0.0218
		Modification-dependent protein catabolic process	6	0.0412
	MF	GTP binding	5	0.0319
(v) Gradual down	BP	Muscle contraction	6	0.0044
	tRNA processing	6	0.0436
	MF	Ion channel activity	9	0.0088
	Structural constituent of ribosome	14	0.0127

BP, biological process; CC, cellular component; MF, molecular function.

**Fig 3:**
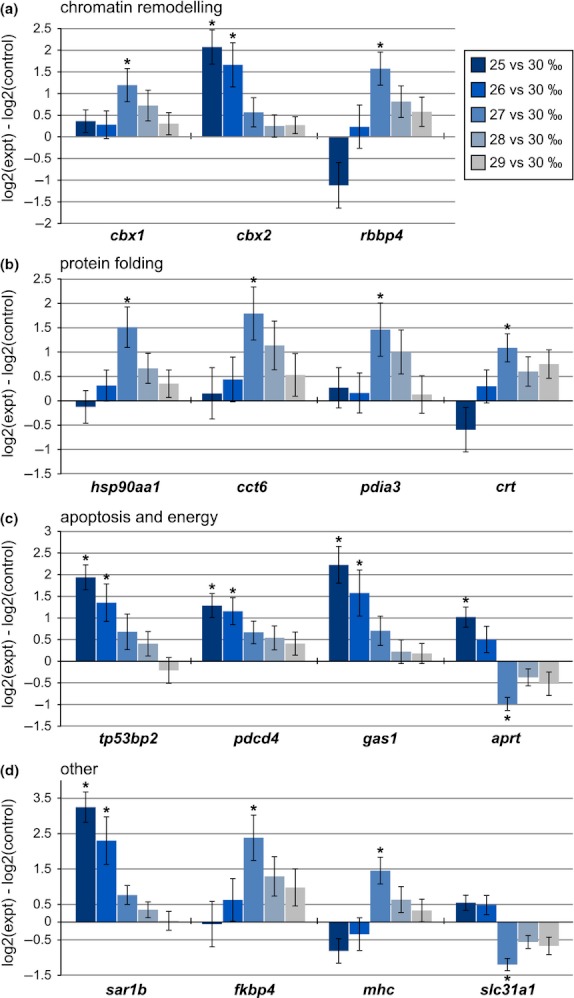
RT–qPCR of selected genes involved in key processes identified by functional enrichment analysis confirms patterns identified in transcript expression clustering. Expression levels are displayed as log_2_ fold change ± SEM for genes of interest (log_2_(experimental) – log_2_(control)). A ratio value of 1 is a 2-fold change, and asterisks denotes significance in difference of condition against control (*P* ≤ 0.05).

Other genes present in the primary response (cluster *i*) are involved in energy acquisition and control (i.e. cell redox homoeostasis, carbohydrate catabolic process) and chromatin binding (Table 4). Chromatin regulation may be involved in the coordination of the responses at different salinities, enabling highly co-regulated suites of genes (Fig. 2). Epigenetic and chromatin regulation is probably important for integrating environmental signals and cell stress with transcriptional programmes (Kim *et al*. 2010). Two chromobox homologs were up-regulated at different phases of the response; *cbx1* followed the primary response, whereas the induction of *cbx2* was consistent with the secondary response (Fig. 3). *Histone-binding protein rbbp4* was up-regulated with *cbx1* at 27 ‰ (Fig. 3). Interestingly, metamorphosis was identified as an enriched GO category in the primary peak cluster, possibly relating to early stages of tissue reorganization in response to hyposaline stress. Heat shock has been shown to induce metamorphosis in some sessile marine invertebrates (Kroiher *et al*. 1992; Gaudette *et al*. 2001). The presence of moulting nauplii in the samples probably contributed to this signature.

Genes that are gradually up-regulated and significantly different from control by 26 ‰ or 25 ‰ (cluster *iv*) are involved in macromolecule catabolism (eight genes; *P* < 0.014) and proteolysis (Table). These functions may be involved in energy acquisition, the degeneration of peptides to generate free amino acids for hypo-osmotic stress buffering, the degradation of accumulated misfolded proteins or a combination of these. Alternatively, genes that are gradually down-regulated by 26 ‰ and 25 ‰ (cluster *v*) included ion channel functions (9 genes; *P* < 0.01), muscle contraction and ribosomal functions (Table 4). Decreased ribosomal functions (including structural constituents of ribosomes and tRNA processing) may relate to protein translation inhibition for energy preservation and/or halting protein production due to accumulated misfolded proteins. Decreased production of structural proteins may be related to the involvement of muscle contraction, although this could also be due to ionic imbalances from hyposaline stress.

Secondary response genes (cluster *iii* in Fig. 2) are involved in transport (Table 4; 45 genes; *P* < 0.01), response to stress (18 genes; *P* = 0.04), small GTPase-mediated signal transduction (14 genes; *P* < 0.001), several remodelling/metamorphosis-related categories and small conjugating protein ligase activity (eight genes; *P* = 0.028). Although several transport proteins were up-regulated at the 27 ‰ response group, such as *Na/K-transporting ATPase subunit alpha* and *sarcoplasmic/endoplasmic reticulum calcium ATPase 1*, the majority of transport proteins are responding at the lower salinities, including down-regulation of several calcium channels and up-regulation of several amino acid transporters and V-type proton ATPase subunits (Table 5). The stress response is probably related to the identified apoptosis-related transcripts up-regulated at 26 ‰ and 25 ‰, such as *apoptosis-stimulating of p53 protein 2* (*tp53bp2*), *programmed cell death protein 4* (*pdcd4*; Fig. 3) and *caspase-1 subunit p12* (Table 6). However, this is not clear, as other transcripts such as up-regulated *bax inhibitor 1* and *fas apoptotic inhibitory molecule 2* indicate anti-apoptotic activity (Table 6). Small GTPase-mediated signal transduction may relate to vesicular transport. Vesicular transport via COPII vesicles is involved in the unfolded protein response (UPR) maintaining ER homoeostasis by regulating endoplasmic reticulum–associated degradation (ERAD) (Higashio & Kohno 2002; Liu & Chang 2008). *GTP-binding protein sar1b* was up-regulated >8-fold at 25 ‰ (Fig. 3) and is an important component of vesicle budding during ER COPII transport (Higashio & Kohno 2002) and cargo proteins transport (Takai *et al*. 2001). Whether the role of this is to alleviate ER stress or to move newly synthesized transport proteins (Table 5) to the cell membrane is unknown. With continual catabolic-related increases (Table 4), down-regulation of protein translation machinery and up-regulation of *growth arrest-specific protein 1* (*gas1*) at 26 ‰ and 25 ‰ (Fig. 3) energy may be a constraint in coping with the abiotic stress. The response at 25 ‰ is more indicative of a stress response, of tissue remodelling (including apoptosis) and of longer-term coping mechanisms compared with the potentially transient response at 27 ‰.

**Table 5:** Hyposalinity affected the expression of genes for transporters of molecules (e.g. amino acids), ions or protons. Relative to the 30 ‰ control, at 25 ‰, calcium transporters were down-regulated, whereas amino acid and proton transporters were mainly up-regulated. Differential expression from the control was tested by one-way anova and Tukey's HSD (*P* ≤ 0.01; FC ≥ 1.5). Fold change ratios are log_2_(experimental) – log_2_(control) with standard error (value of + 1 = 2-fold up-regulation). Absent values indicate no significant difference from control

Function	Gene	Probe ID	Salinity (‰)				
			25	26	27	28	29
Ion – Sodium & Potassium	Bumetanide-sensitive sodium-(potassium)chloride cotransporter	C215R132	1.00 ± 0.15	–	0.88 ± 0.19	–	–
	Sodium/potassium-transporting ATPase subunit alpha	C006R049	–	–	0.59 ± 0.18	–	–
	Sodium/potassium-transporting ATPase subunit alpha-1	C214R147	−1.24 ± 0.20	–	–	–	–
	Trimeric intracellular cation channel type A	C112R134	−1.97 ± 0.34	−1.30 ± 0.44	–	–	–
	Trimeric intracellular cation channel type B	C145R152	−0.62 ± 0.18	−0.83 ± 0.18	−0.76 ± 0.16	–	–
Ion - Calcium	Voltage-dependent calcium channel type D subunit alpha-1	C071R127	−0.76 ± 0.08	−0.91 ± 0.18	–	–	–
	Plasma membrane calcium-transporting ATPase 1	C121R156	−1.59 ± 0.37	–	–	–	–
	Plasma membrane calcium-transporting ATPase 2	C233R020	−2.05 ± 0.21	−1.46 ± 0.51	–	–	–
	Calcium channel flower	C016R093	−0.92 ± 0.17	–	–	–	–
	Sarcoplasmic/endoplasmic reticulum calcium ATPase 1	C201R145	–	–	1.28 ± 0.25	–	–
Calcium-binding protein p22	C229R056	0.99 ± 0.21	1.09 ± 0.29	–	–	–
Sarcoplasmic calcium-binding protein, beta chain	C263R153	–	0.80 ± 0.15	–	–	–
Ammonium	Ammonium transporter Rh type B-B	C057R148	−1.31 ± 0.19	−1.11 ± 0.13	−1.17 ± 0.27	–	–
	Ammonium transporter Rh type C	C065R149	−1.19 ± 0.17	−0.92 ± 0.18	−1.19 ± 0.30	–	–
Amino acid	Proton-coupled amino acid transporter 4	C203R034	1.62 ± 0.28	1.71 ± 0.38	–	–	–
	Low-affinity cationic amino acid transporter 2	C203R001	1.26 ± 0.29	–	–	–	–
	Orphan sodium- and chloride-dependent neurotransmitter transporter NTT73	C094R122	−1.79 ± 0.33	−1.42 ± 0.31	–	–	–
Proton (pH)	V-type proton ATPase 16 kDa proteolipid subunit	C061R055	1.34 ± 0.16	–	–	–	–
	V-type proton ATPase subunit C	C073R064	1.60 ± 0.29	–	–	–	–
	V-type proton ATPase subunit D	C107R139	0.61 ± 0.14	–	–	–	–
	V-type proton ATPase subunit E	C011R121	1.05 ± 0.21	–	–	–	–
	V-type proton ATPase subunit e 2	C046R143	1.43 ± 0.22	–	–	–	–
	V-type proton ATPase subunit F	C190R101	1.59 ± 0.19	1.25 ± 0.32	–	–	–

**Table 6:** Hyposalinity affected the expression of genes involved in apoptosis (programmed cell death) and acid/base balance and detoxification. Relative to the 30 ‰ control, many of these functions were up-regulated at 25 ‰. Differential expression from the control was tested by one-way anova and Tukey's HSD (*P* ≤ 0.01; FC ≥ 1.5). Fold change ratios are log_2_(experimental) – log_2_(control) with standard error (value of + 1 = 2-fold up-regulated). Absent values indicate no significant change from control

Function	Gene	Probe ID	Salinity (‰)				
			25	26	27	28	29
Apoptosis	Apoptosis-stimulating of p53 protein 2	C179R103	1.04 ± 0.19	0.80 ± 0.21	–	–	–
	Autophagy-related protein 16-1	C037R044	–	−0.86 ± 0.19	–	–	–
	Caspase-1 subunit p12	C225R096	1.45 ± 0.42	1.62 ± 0.38	–	–	–
	Fas apoptotic inhibitory molecule 2	C120R093	1.32 ± 0.20	1.06 ± 0.29	–	–	–
	Programmed cell death protein 4	C168R058	1.63 ± 0.26	1.26 ± 0.30	–	–	–
	Bax inhibitor 1	C233R167	1.30 ± 0.37	–	–	–	–
	Growth arrest-specific protein 1	C222R122	1.50 ± 0.34	–	–	–	–
Acid/base balance & detoxification	Beta-carbonic anhydrase 1	C212R074	1.28 ± 0.33	–	–	–	–
	Glutathione S-transferase kappa 1	C038R059	–	–	−0.69 ± 0.16	–	–
	Microsomal glutathione S-transferase 1	C190R083	–	–	–0.84 ± 0.13	–	–
	Glutathione S-transferase DHAR1, mitochondrial	C208R018	1.01 ± 0.13	–	–	–	–
	Glutathione S-transferase Mu 3	C102R105	0.86 ± 0.18	–	–	–	–
	Glutathione S-transferase kappa 1	C038R059	–	–	−0.69 ± 0.16	–	–

### Correlation between qPCR and microarray

Microarray expression levels correlated well with qPCR expression levels (Figure S1, Supporting information). *R*^2^ values and slope from the best fit line of each sample's log_2_ expression value from qPCR against microarray are displayed in Figure S1 (Supporting information) (average (and median) of *R*^2^ and slope values for genes in Fig. are 0.70 (0.68) and 0.82 (0.74), respectively). The clusters were confirmed through the RT–qPCR analysis, including the primary peak, primary valley and secondary response (Fig. 3). Only *aquaporin-9*,*hsp90 co-chaperone cdc37* and *collagen alpha-2 (IV) chain* of 18 tested genes did not show similar patterns (*not shown*), possibly due to the amplification of paralogs or to false positives from microarray results.

## Discussion

A relatively brief hyposaline exposure resulted in large transcriptional changes consistent with distinct stress responses in larval dispersal stages of *L. salmonis*. In contrast, a similarly large effect on transcription was not observed following short-term exposures to hypo- or hyperthermal environments, although some effects were identified at high temperature. It is possible that longer-term exposures (days–weeks) to hypothermal environments would have a larger effect on growth-related functions. Experimental replication with different broods of lice indicated variation in responses which may partly result from differences in energy reserves among individuals (Bricknell *et al*. 2006). Despite this variation, it is clear that hyposaline water causes large-scale changes in gene expression programmes of *L. salmonis* larvae.

Host-seeking behaviour displayed by *L. salmonis* includes movement towards and maintenance at haloclines near river mouths during salmon migrations, and thus, copepodids must be able to cope with short-term salinity fluctuations (Brooks 2005). Coping mechanisms are expected to minimize effects of suboptimal environments and to minimize costs associated with coping. The transient cellular stress response (CSR) is induced by various stressors through macromolecule damage and can target cell cycle control, protein chaperoning, DNA/chromatin stabilization, removal of damaged proteins and some aspects of metabolism (reviewed in Kültz 2005). The threshold response at 27 ‰ may be an *L. salmonis* CSR, characterized by chaperone and proteasome activity, chromatin binding and redox homoeostasis (Table 3,4; Fig. 3). Down-regulated genes at 27 ‰ (cluster *ii*; Fig. 2), including structural components of ribosomes (Table 4), may indicate the down-regulation of other genes during rapid onset of Hsps (Rinehart *et al*. 2007). Proteasome and chaperone activities usually require ATP hydrolysis (Kültz 2005), and therefore, this coping strategy requires energy expenditure. Below 27 ‰, the initial suite of chaperones may not be optimal for the level of stress (possibly due to elevated energy consumption), as the expression of the Hsps responding at 27 ‰ is at baseline in the 26 ‰ condition (Table 3). It is also possible these chaperones are not up-regulated at salinities <27 ‰, because a second suite of chaperones are better suited to the less transient stress (Table 3) or because of anti-apoptotic activity of chaperones (see Kültz 2005 for review).

The coping strategy at 26 ‰ and 25 ‰ involves up-regulation of transporters, apoptosis-related genes, different types of chaperones (Table 3) and genes involved in vesicular transport (Fig. 3). A less-rapidly induced programme of cells is the cellular homoeostatic response (CHR), a long-term process that will continue until the stressor is removed (Kültz 2005). Unlike the aforementioned CSR, the CHR is stressor specific, with sensors specific to the environmental change (Kültz 2005). The response at 25 ‰ may be typical of a *L. salmonis* CHR to low salinity. Aspects of this response are similar to osmoregulation. Expression changes of some transporters (Table 5) are similar to those identified in the gills of the euryhaline green crab *Carcinus maenas* responding to hyposalinity (Towle *et al*. 2011). For example, *Na*^*+*^*/K*^*+*^*-ATPase alpha subunit* and *carbonic anhydrase* were up-regulated in the green crab at 10–15 ‰ (Towle *et al*. 2011) and also in the present study at 27 ‰ and 25 ‰, respectively (Table 5, 6). Vesicular transport was identified as an important part of the 25 ‰ response in the present study (Table 4; Fig. 3), and a gene involved in regulating plasma membrane protein composition was up-regulated in the green crab in a hypo-osmotic environment (Towle *et al*. 2011). Movement of transporter proteins to cell membranes is important for cellular osmoregulation to increase activity of certain ion pumps and amino acid transporters for pumping free amino acids out of the cell to buffer the osmotic gradient between the cell and interstitial spaces (Pierce 1982). However, differences between the response of the euryhaline green crab gills and *L. salmonis* copepodids, including stable expression of stress-related transcripts in the green crab gill (e.g. HSPs, proteasome subunits) that were up-regulated in louse copepodids along with several apoptotic transcripts (Table 3, 6; Fig. 3), may be attributed to the euryhaline nature of the crab (Towle *et al*. 2011) compared with the stenohaline copepod. Differences in expression changes of voltage-gated calcium channels and the stable expression of V-type H^+^-ATPase in the green crab also differed from the present study, in which multiple subunits were found up-regulated at 25 ‰ (Table 5). The V-type H^+^-ATPase was shown to be important for hypo-osmotic regulation in the marine copepod *Eurytemora affinis* (Lee *et al*. 2011). While differences in tissue profiling (crab gill vs. whole copepod) should be noted, the similarities and differences in patterns of gene expression displayed by the green crab and *L. salmonis* copepodids highlight the relative sensitivity of free-swimming lice to a hyposaline stressor.

Although these coping mechanisms appear necessary for survival of *L. salmonis*, the energetic costs are probably significant for nonfeeding life stages. Increased expression of catabolic process transcripts at 25 ‰ suggests the high cost of these long-term coping strategies (Table). Highly up-regulated *sar1b* expression at 25 ‰ (Fig. 3) suggests coordination of the unfolded protein response and vesicular transport, alleviating endoplasmic reticulum stress caused by accumulated misfolded proteins (Higashio & Kohno 2002). If stress exceeds tolerance limits, the result of individual cells is growth arrest and apoptosis (Kültz 2005), which may be occurring in *L. salmonis* at 25 ‰ (Table 6; Fig. 3c). The alternative of these costly mechanisms, and the ultimate outcome once energy reserves are depleted, is probably organism death, as was viewed in 50% of copepodids (Atlantic) after 1 h at 16 ‰ salinity (Bricknell *et al*. 2006).

The regulation of a multitude of genes is being affected by hyposalinity (Fig. 2), and this may be enabled through chromatin remodelling (Table 4; Fig. 3a). Plant responses to environmental stress, such as drought, are integrated and coordinated through histone modifications, changes in nucleosome occupancy, DNA methylation changes and other chromatin remodelling methods (Kim *et al*. 2010).

The sensitivity of *L. salmonis* copepodids (Pacific) to hyposalinity is indicated by the increased expression of coping-related transcripts after 24 h at 27 ‰ seawater and by larger changes in expression profiles identified at 26 ‰ and 25 ‰ seawater. It will be important to determine whether these patterns of response to hyposalinity differ between *L. salmonis* varieties occurring in the Pacific and Atlantic Oceans (Yazawa *et al*. 2008). The results of this work may assist in the interpretation of salinity maps of coastal zones by identifying areas in which larval *L. salmonis* are likely to survive or experience hyposalinity-associated stress. Although adult forms can be more robust to hyposalinity stress, it is important to consider juvenile forms when defining optimal environmental ranges (Lockwood & Somero 2011). Further, as suggested by Brooks (2009), if levels of lice are not higher than set thresholds and a freshwater influx is expected, treating after the natural stressor may be best to reduce numbers of chemical treatments to reduce environmental residues and slow down the development of resistance. Although this work may be useful for salmon farm location identification, some areas with large freshwater inputs may not be suitable as aquaculture sites due to the importance of preserving wild migratory routes (Johnson *et al*. 2004; Krkošek *et al*. 2007; Jones *et al*. 2008; Sutherland *et al*. 2011). Regardless of extent of population-level effects, the present work indicates the importance of monitoring salinity around salmon farms.

## Conclusions

A short-term (24 h) exposure to hyposalinity elicited significant changes to the transcriptome of free-swimming larval *Lepeophtheirus salmonis*. These changes were indicative of short- and long-term coping strategies adopted by the copepod that varied according to the extent of hyposaline stress and potentially the energy reserves of the louse. Transient strategies used ATP-dependent molecular chaperones to maintain cellular integrity, whereas longer-term strategies used transporters and channels in combination with different chaperones. Short-term (24 h) temperature exposures between 10 and 4 °C did not result in major changes in transcription. Elevated temperature (16 °C) affected louse transcriptome profiles, although not to the same extent as was viewed in salinity exposures. Despite variable responses among experimental replicates, consistent patterns were identified, and this work provides stressor-level and stressor-type context for ecological response genes.

## Supporting Information

Fig S1 Correlation between log2 qPCR (y-axis) and log2 microarray (x-axis) expression values.

Table S1 Response genes common between wide-range experimental replicates.

Table S2 Probes present in each cluster in high-resolution salinity experiment.
