# Supplementary figures and images for: Transcriptomics of coping strategies in free-swimming Lepeophtheirus salmonis (Copepoda) larvae responding to abiotic stress
Source: Mol Ecol. 2012 Oct 25;21(24):6000–14. doi: 10.1111/mec.12072 (PMC3557717; doi:10.1111/mec.12072)

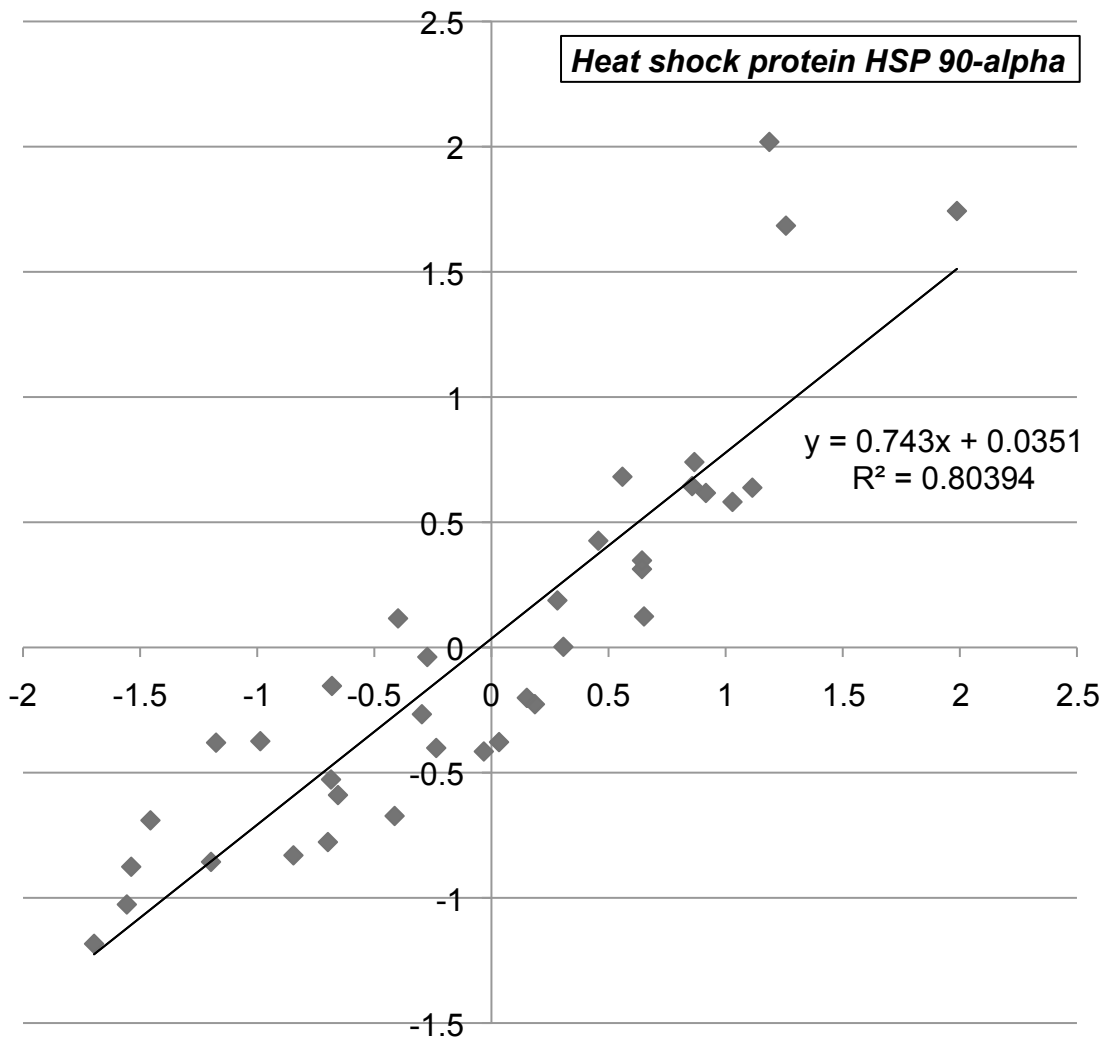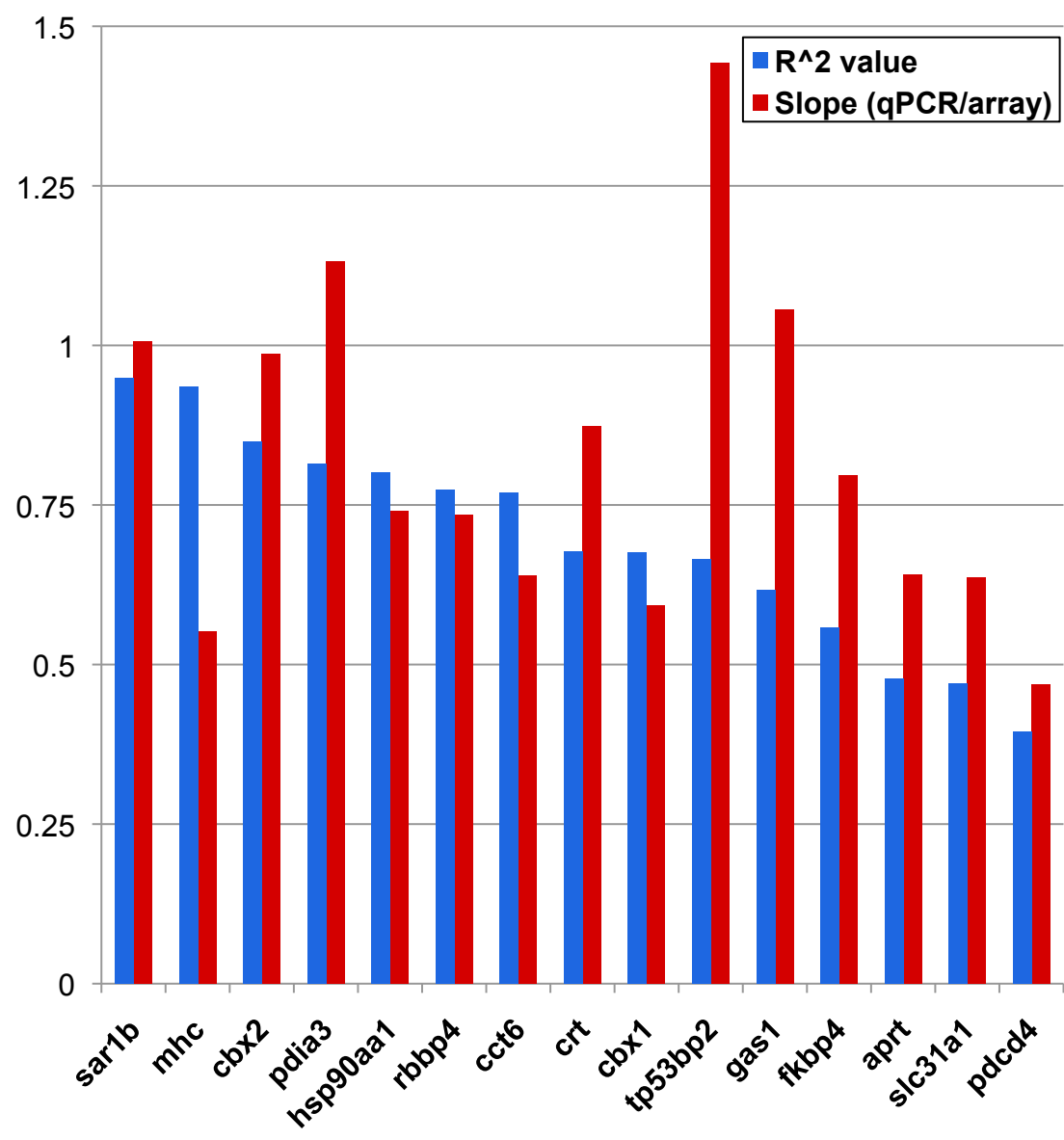

Supplement: Fig S1 — Correlation between log2 qPCR (y-axis) and log2 microarray (x-axis) expression values. [file mec0021-6000-sd1.pdf]
